# Fluctuations of sensorimotor processing in migraine: a controlled longitudinal study of beta event related desynchronization

**DOI:** 10.1186/s10194-019-1026-8

**Published:** 2019-07-09

**Authors:** Martin Syvertsen Mykland, Marte Helene Bjørk, Marit Stjern, Petter Moe Omland, Martin Uglem, Trond Sand

**Affiliations:** 1 Department of Neuromedicine and Movement Science, Faculty of Medicine and Health Sciences, NTNU - Norwegian University of Science and Technology, Trondheim, Norway; 2 Department of Clinical Medicine, University of Bergen, Bergen, Norway; 3 Department of Neurology, Haukeland University Hospital, Bergen, Norway; 4 Department of Neurology and Clinical Neurophysiology, St. Olavs Hospital, Trondheim, Norway

**Keywords:** Migraine, Headache, Cycle, Cortex, Thalamus, EEG, ERD, Pre-activation, Pathophysiology, Neurophysiology

## Abstract

**Background:**

The migraine brain seems to undergo cyclic fluctuations of sensory processing. For instance, during the preictal phase, migraineurs experience symptoms and signs of altered pain perception as well as other well-known premonitory CNS-symptoms. In the present study we measured EEG-activation to non-painful motor and sensorimotor tasks in the different phases of the migraine cycle by longitudinal measurements of beta event related desynchronization (beta-ERD).

**Methods:**

We recorded electroencephalography (EEG) of 41 migraine patients and 31 healthy controls. Each subject underwent three EEG recordings on three different days with classification of each EEG recording according to the actual migraine phase. During each recording, subjects performed one motor and one sensorimotor task with the flexion-extension movement of the right wrist.

**Results:**

Migraine patients had significantly increased beta-ERD and higher baseline beta power at the contralateral C3 electrode overlying the primary sensorimotor cortex in the preictal phase compared to the interictal phase. We found no significant differences in beta-ERD or baseline beta power between interictal migraineurs and controls.

**Conclusion:**

Increased preictal baseline beta activity may reflect a decrease in pre-activation in the sensorimotor cortex. Altered pre-activation may lead to changes in thresholds for inhibitory responses and increased beta-ERD response, possibly reflecting a generally increased preictal cortical responsivity in migraine. Cyclic fluctuations in the activity of second- and third-order afferent somatosensory neurons, and their associated cortical and/or thalamic interneurons, may accordingly also be a central part of the migraine pathophysiology.

## Introduction

Migraine patients undergo transient clinical and neurophysiological changes before, during and after headache attacks. These intervals define the phases of the migraine cycle, and pain perception changes transiently between the phases [1, 2]. In the days and hours preceding headache, various other symptoms including yawning, nausea, changes in mood and activity, fatigue and neck symptoms emerge [3–9]. Furthermore, neuroimaging and neurophysiological studies show alterations of the central nervous system (CNS) preceding the eventual aura and the pain [2, 10–20]. Sensitized thalamic neurons may mediate allodynia and hyperalgesia [8, 21], and the connectivity between thalamus and several brain regions has also been shown to undergo cyclic changes in migraineurs [22]. However, there is disagreement between different studies regarding whether the migraine brain become hypo- or hyperexcitable [19, 23–25]. Further mapping of the preictal neurophysiological state may reveal the probable CNS generators for the migraine attacks.

One technique for measuring cortical activation and increased excitability during sensorimotor processing is beta event-related desynchronization (beta-ERD) [26–28]. Event related desynchronization and synchronization (ERD/ERS) are electrophysiological features that represent an induced, time-locked, non-phase-locked response to events. These responses are subject to changes in neuronal synchrony. Furthermore, they are specific for frequencies in the electroencephalography (EEG). In general the ERD/ERS is understood to represent changes in activity of the interactions between thalamocortical networks and cortical interneurons [29]. The cortical activity accompanying voluntary limb movement is for instance represented by ERD of alpha and beta bands in the contralateral sensorimotor cortex, reflecting cortical activation with enhanced information processing [27, 28]. More recent knowledge of time averaged beta-ERD suggest that the strength of the ERD response during hand movements represents mostly the afferent proprioceptive sensation and sensory processing [30]. One study of fibromyalgia patients showed altered beta-ERD in response to tactile stimulation, which was interpreted by the authors as physiological changes that contribute to chronic pain in this patient group [31]. This technique may accordingly be useful for investigating cortical sensory processing in migraine patients.

As the migraine brain is subject to cyclic neurophysiological fluctuations, it is advantageous to conduct longitudinal studies [32]. In a recent paper on post-movement beta synchronization (PMBS) in migraine [14], we revealed cyclic fluctuations of post-stimulation inhibition in sensorimotor cortex. To our knowledge the actual desynchronization during movement has not been analysed previously in migraine. Thus, in the present paper we aimed to measure thalamocortical excitability in migraine patients by cortical beta-ERD during sensory processing of afferent inputs from hand movements. The first primary aim was to compare cyclic changes from the interictal baseline to the preictal, ictal and postictal phases. The second primary aim was to compare interictal beta-ERD between migraineurs and healthy controls. A third, and secondary, aim was to evaluate if these electrophysiological responses correlated with clinical symptoms and severity.

## Subjects and methods

### Subjects

The general methodology for this study is described in our previous papers [14, 18–20, 32, 33]. We recruited episodic migraine patients by a newspaper advertisement, screening by trained nurses, and evaluation for inclusion by a neurologist [32]. They had 2–6 migraine attacks each month. Healthy controls were recruited among blood donors. Exclusion criteria were frequent episodic or chronic tension-type headache, acute or chronic disease, pregnancy, alcohol or drug abuse, neuroactive drug use and migraine prophylactic drugs within four weeks before the test. We included 41 migraine patients and 31 healthy controls. All but 4 patients and 3 controls were right handed.

We recorded demographic data on all subjects in addition to clinical presentation of the migraine patients by a questionnaire and a semi-structured interview (Table 1). Every migraine patient also completed a headache diary from 2 weeks before inclusion until 2 weeks after the last EEG recording. This enabled us to classify the headache and its temporal relationship to the EEG recordings as preictal (< 36 h before attack), postictal (< 36 h after attack), ictal (pain attack) and interictal (> 36 h from attack). The choice of 36 h cut off is based on an electronic diary study revealing that reliable premonitory symptoms mainly occurred within a one-day cut off [7], and the utility of the 36 h cut off used in our previous EEG-studies in migraine [14, 18–20, 32, 33]. Every subject (except one) had three EEG recordings at the same time of day with 3–10 days intervals**.** Thirty-three patients had at least one interictal recording. Patients with both near-attack and interictal recordings formed three additional migraine subgroups for intraindividual paired analysis (Table 1). Six patients had visual aura, two of whom with additional somatosensory aura.

**Table 1:** Demographic and clinical data on groups used in interictal analysis (comparing recordings in interictal migraineurs and controls) and subgroups used in paired analysis

		Migraineurs for paired analysis^2^		
	Interictal recordings^1^	Preictal	Ictal	Postictal
	(*n* = 33)	(*n* = 11)	(*n* = 13)	(*n* = 9)
Women/men	30/3	11/0	12/1	7/2
MwoA/MA	27/6	9/2	10/3	8/1
Age (years)	36.5 (12.7)	37.3 (12.9)	37.5 (12.5)	41.3 (12.8)
Headache history (years)	19.3 (11.0)	20.5 (11.7)	20.5 (9.9)	18.1 (13.1)
Headache days last 3 months	6.2 (4.0)	6.7 (4.8)	7.2 (4.7)	4.2 (2.3)
Headache intensity (0–4)	2.4 (0.7)	2.4 (0.7)	2.3 (0.6)	2.2 (1.0)
Headache duration (h)	17.8 (22.0)	15.9 (20.2)	14.9 (17.3)	18.4 (30.5)
Photophobia (0–2)^3^	1.4 (0.7)	1.4 (0.7)	1.0 (0.8)	1.2 (0.7)
Phonophobia (0–2)^3^	1.1 (0.8)	1.2 (0.7)	0.8 (0.8)	1.2 (0.8)

*MA* = migraine with aura, *MwoA* = migraine without aura. Mean (SD) or numbers

^1^Migraineurs with at least one interictal EEG recording

^2^Subgroups with both an interictal EEG recording (> 36 h from attack) and a preictal (< 36 h before attack), ictal or postictal (< 36 h after attack) EEG recording

^3^Numerical definition: 0 – none, 1 – some, 2 – substantial

The control group consisted of 27 women and 3 men (*n* = 30) with a mean age of 39.7 (11.5)

Staff involved in data recording and EEG-response processing was blinded to the diagnosis status. The subjects received NOK 1000 (about EUR 103 with current exchange rates) as compensation to cover expenses after completing all three recordings. The compensation was not mentioned in the advertisement.

### EEG recordings and experimental setup

We recorded approximately 30 min EEG with eyes closed. Five minutes undisturbed relaxed wakefulness was followed by a motor and a sensorimotor test and by photic stimulation. We have previously reported resting state quantitative EEG, steady-state visual evoked potentials and PMBS [14, 18, 19, 32, 33].

**Fig. 1:**
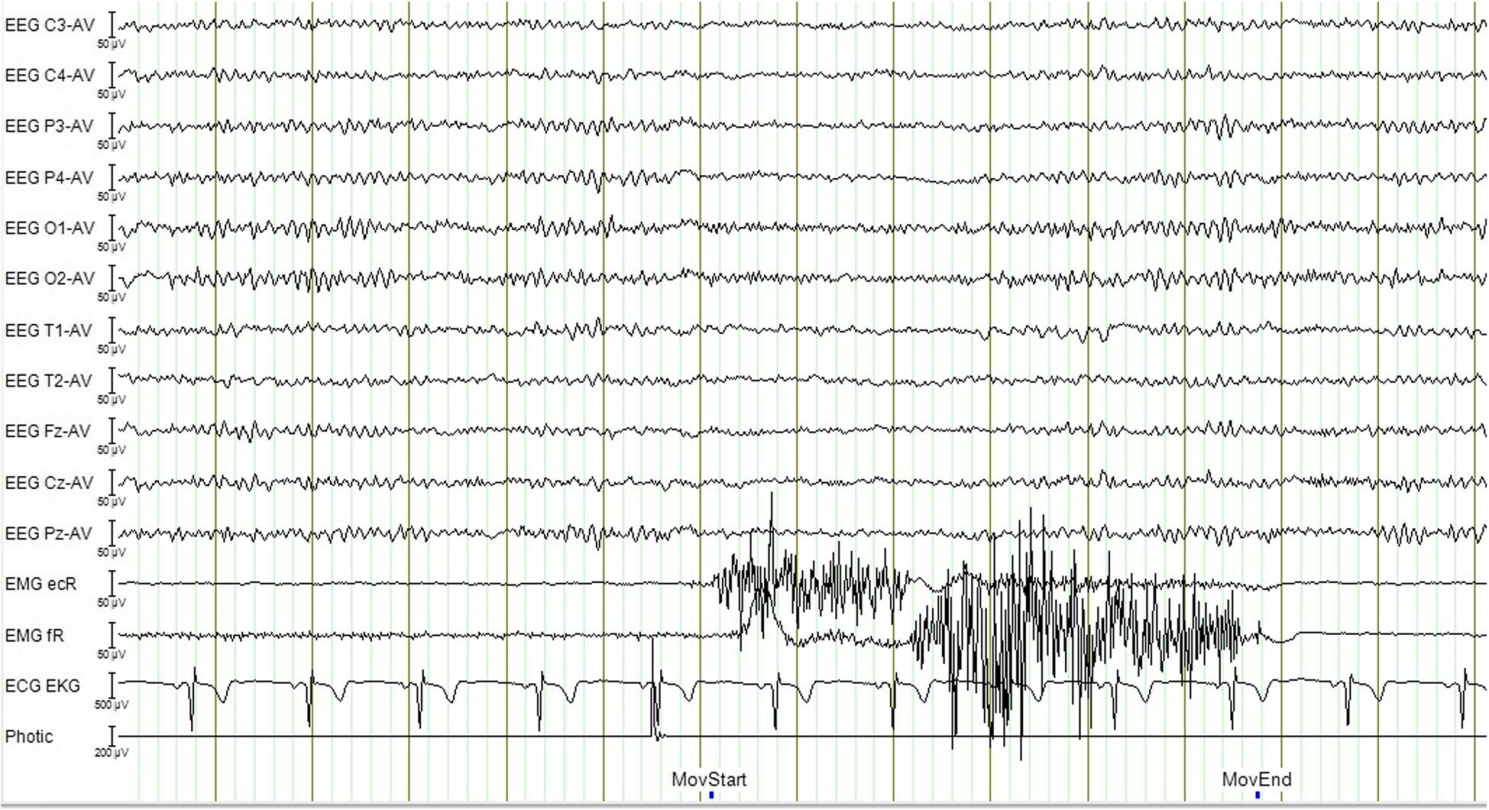
EEG, EMG (ecR: radial wrist extensors, fR: forearm wrist flexors), ECG and photic channels from recording of one single movement. MovStart and MovEnd markers indicate start and end of movement as shown by EMG channels. The photic channel marker represents a light blink as a sign for the subject to prepare for executing the task. EEG channels visually revealing desynchronization from start of movement

We attached twenty-four scalp electrodes according to the 10/20 international system [34] with channels for lateral anterior temporal electrodes, horizontal and vertical eye movements, and ECG. EEG was recorded digitally in Nervus 3.0 with M40 amplifier (Natus Medical Inc., Pleasanton, CA 94566, USA) and common reference with 256 Hz sampling rate. We used an average reference montage with low- and high-pass filter of 0.5 and 70 Hz in addition to notch filter (50 Hz). Two EMG-channels for flexion and extension were included in the EEG recording for determination of movement epochs (Fig. 1).

Each subject performed both a motor test (M) and a sensorimotor test (SM) with approximately 30 repeated movements of the right arm in each test. The order of tests was randomized for each subject and fixed for each day of recording for the same subject. The instructions given to all subjects were the following: Each test would last about 8 min with a light blink indicating when to start each movement. For the motor test, subjects were to first flex their wrist for 2 s, then extend their wrist for 2 s, followed by about 15 s of relaxation. For the sensorimotor test an identical flexion-extension movement supplemented by a discrimination task was performed: A bowl of different material spheres (wood or metal) was placed about 5 cm below the neutrally positioned fingers so that the fingers were in contact with the spheres in the flexed position. The task was to use the 2 s in the flexed position to scan spheres lightly with their fingertips to detect if a sphere of wood was present in the bowl or not. The right arm was used in both sequences.

### ERD analysis

**Fig. 2:**
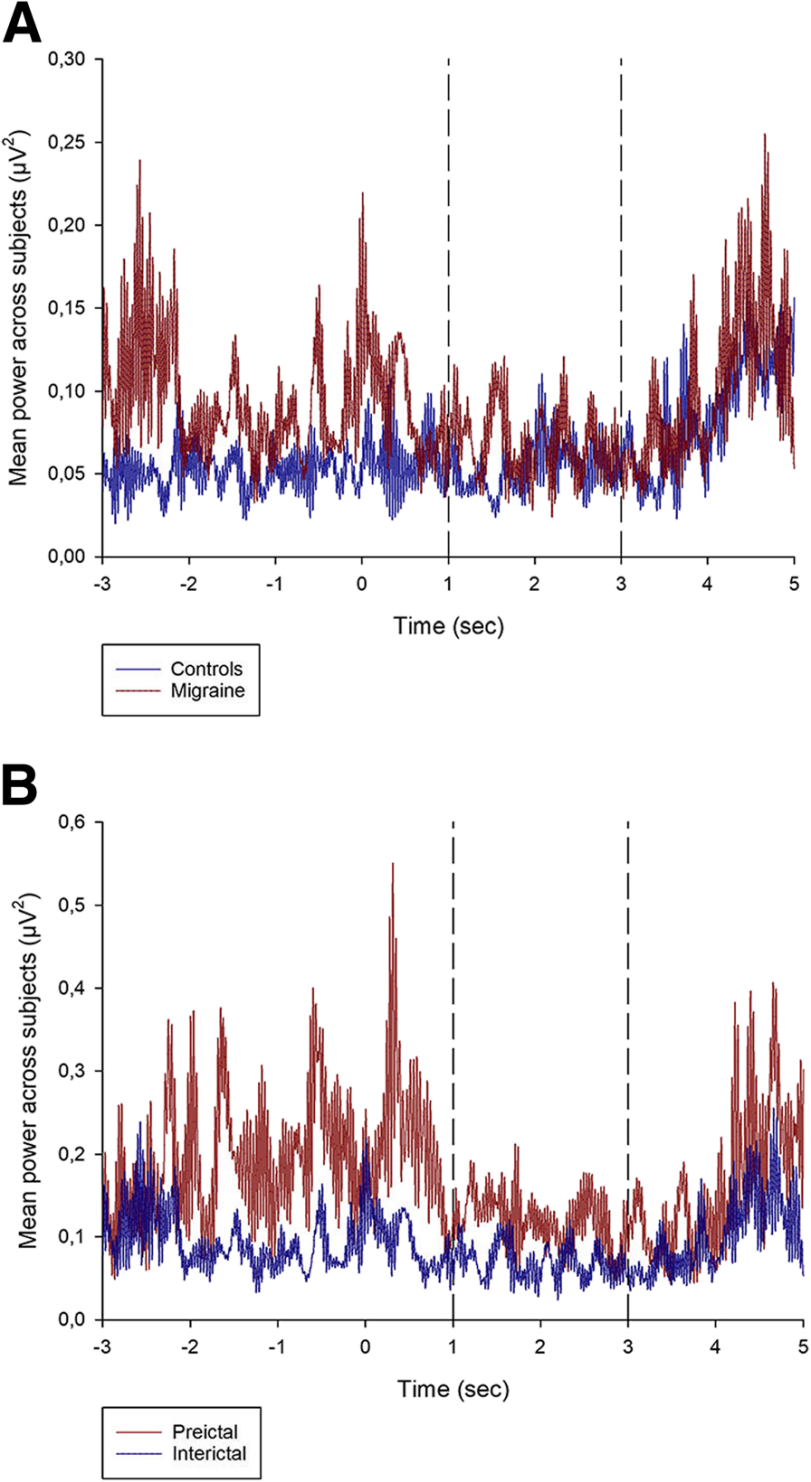
**a** Grand mean power across subjects at the contralateral C3 electrode for the sensorimotor task in controls and interictal migraine patients. **b** Grand mean power across subjects at the contralateral C3 electrode for the sensorimotor task in migraine patients for interictal and preictal recordings. First two seconds (−3 to − 1) represent pre-movement onset baseline. 0 represent start of movement. Broken vertical lines indicate the selected interval (1 to 3 s) for the ERD period

IIR-filtered data in the 12–19 Hz beta frequency band [35, 36] from sensorimotor cortices electrodes C3 (left side, contralateral) and C4 (right side, ipsilateral), were exported in 256 Hz resolution from each test and used for the beta-ERD analysis. Voluntary movement intent lowers beta band activity close to the contralateral sensorimotor cortical area, and this beta-ERD spreads and becomes bilateral right before movement execution [29, 37, 38]. We averaged the squared EEG-amplitude across all movements within the same test [29]. Movement onset and offset were marked (Fig. 1). We used the EEG-segment from − 3 s to − 1 s (prior to start of movement) as baseline and the EEG-segment from 1 s to 3 s after movement onset for ERD-calculation as established in previous studies [27, 31]. ERD was thereafter calculated as the ratio of power in the activity period to power in the baseline period [27]. ERD can be observed as reduced EEG-amplitude (Fig. 1) and as reduced beta-power during movement (Fig. 2).

### Statistical analysis

For each migraine patient we selected one test for each cyclic phase that was available (interictal, preictal, ictal and postictal). If several tests for the same phase were available, we chose the second one. We selected control EEGs with a similar test-order distribution as the interictal migraine group.

We used paired Student’s t-tests for the three primary pre-planned intrasubject contrasts. Two-sided *p*-values < 0.05 were regarded as significant, and p-values < 0.10 were regarded as trends. A paired analysis with 11 pairs has approximately 77% power to detect an effect = 90% of group SD [32].

To evaluate the combined effects of “phase”, “task” and “side” factors, we also performed three separate repeated measures ANOVAs (R-ANOVA) within the migraine group (preictal-interictal, ictal-interictal and postictal-interictal differences respectively). Three within-subject factors were specified; “phase”, “side” (C3 vs C4) and “SM/M” (sensorimotor vs motor test).

Similarly, to evaluate differences between controls and migraineurs in the interictal phase, we used two sample Student’s t-test followed by R-ANOVA on LN-transformed ERD-ratios. We used within-subject factors “side” (C3 vs C4) and “SM/M” (sensorimotor vs motor test), and between-subjects factor “group” (CO vs MIG).

We calculated exploratory Spearman rho correlations for headache history duration, usual attack duration, individual scores on headache frequency (0–4), usual headache intensity (0–4), individual scores on photophobia (0–2) and individual scores on phonophobia (0–2), and beta-ERD.

## Results

Responses were generally larger (lower ratios) for SM than for M-tasks (Table 2), and the task-factor was significant both in preictal and ictal phases (Table 3).

**Table 2:** Beta-ERD responses for preictal-interictal, ictal-interictal and postictal-interictal comparisons

Beta-ERD response			
	Mean ratio (± SD retransformed)		
C3 Sensorimotor	Interictal	Compared period	p
Preictal	0.84 (0.61–1.15)	0.71 (0.46–1.10)	**.038**
Ictal	0.79 (0.62–1.01)	0.74 (0.54–1.02)	.49
Postictal	0.74 (0.49–1.12)	0.73 (0.54–1.00)	.97
C3 Motor			
Preictal	1.12 (0.78–1.61)	0.96 (0.76–1.21)	**.049**
Ictal	1.05 (0.72–1.52)	1.04 (0.69–1.55)	.93
Postictal	0.93 (0.49–1.74)	0.73 (0.47–1.14)	.23
C4 Sensorimotor			
Preictal	0.83 (0.65–1.06)	0.71 (0.48–1.05)	.23
Ictal	0.83 (0.56–1.21)	0.79 (0.51–1.24)	.70
Postictal	0.68 (0.46–1.02)	0.72 (0.57–0.92)	.72
C4 Motor			
Preictal	1.10 (0.70–1.70)	1.14 (0.74–1.74)	.76
Ictal	1.19 (0.76–1.85)	1.23 (0.82–1.86)	.73
Postictal	1.06 (0.57–1.99)	0.76 (0.44–1.34)	.23

Beta-ERD response is the ratio between mean power in the interval from 1 to 3 s after movement onset and mean power in the interval − 3 to − 1 s before movement onset (baseline). Ratios were LN-transformed before statistical analysis and retransformed to mean ratios and mean ± SD for tabulation. Paired Student’s t-tests are included. EEG from central electrodes C3 (contralateral, left) and C4 (ipsilateral right) for the sensorimotor test and motor test

Bold entries are significant *p*-values

**Table 3:** Repeated measures ANOVA beta-ERD paired analysis in different phases of the migraine cycle (preictal, ictal and postictal; compared to a paired interictal recording)

Beta-ERD response						
Within subjects effects	Preictal		Ictal		Postictal	
	F (1, 10)	p	F (1, 12)	p	F (1, 8)	p
Phase	3.472	**.089**	.045	.84	.833	.39
Side	.319	.58	3.318	**.094**	.162	.70
SM/M	6.084	**.031**	18.706	**.001**	2.623	.14
Side × SM/M	1.231	.29	3.448	**.088**	3.156	.11
Side × Phase	3.105	.11	.533	.48	.094	.77
SM/M × Phase	.539	.48	.305	.59	1.721	.23
Side × SM/M × Phase	2.533	.14	.125	.73	2.072	.19

Paired analysis of subgroups preictal (< 36 h before migraine pain attack), ictal and postictal (< 36 h after migraine pain attack). Beta-ERD response is the ratio between mean power in the interval from 1 to 3 s after movement onset and mean power in the interval − 3 to −1 s before movement onset (baseline). Ratios were LN-transformed before statistical analysis. Factors used were side (C3 vs C4 electrode), SM/M (sensorimotor vs motor) and cyclic phase (preictal-interictal, ictal-interictal and postictal-interictal)

### Paired analyses for preictal, ictal and postictal phases compared to the interictal period

**Fig. 3:**
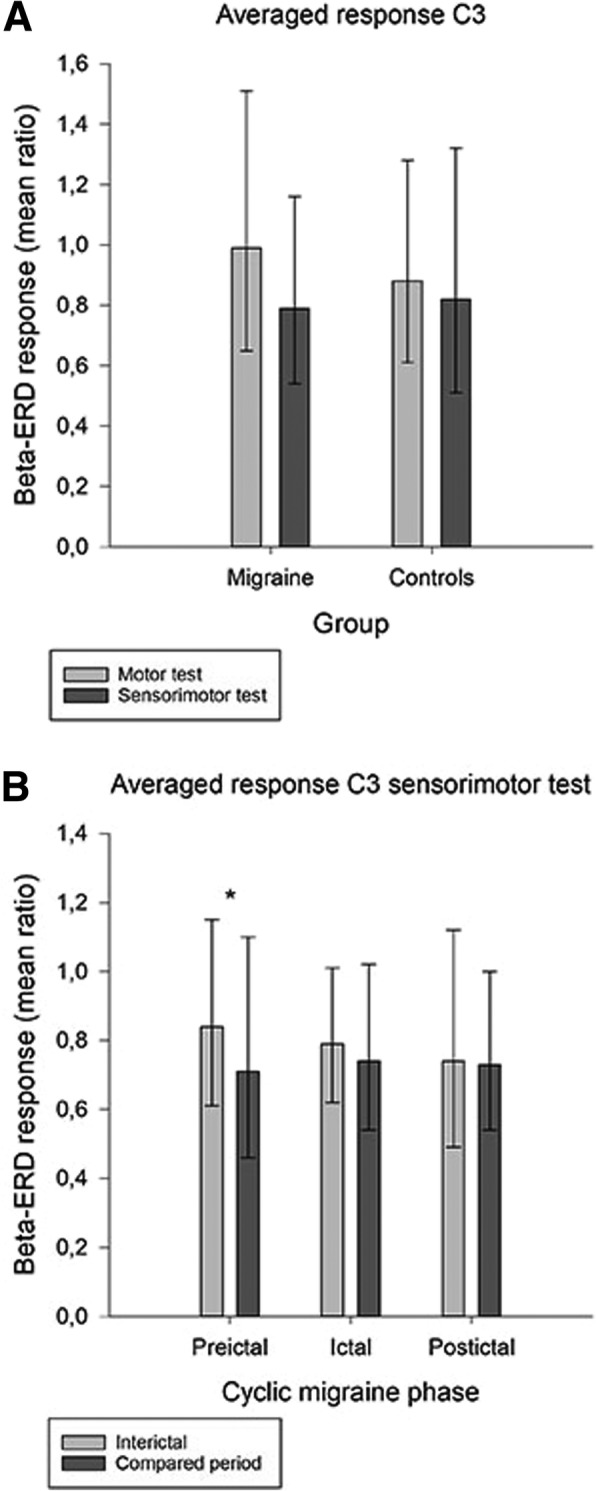
Beta-ERD ratios (movement interval divided by baseline interval) for contralateral sensorimotor cortex (C3) with lower ratios indicating increased ERD-response. Error bars are mean ± SD (retransformed). **a** The figure is illustrating generally larger beta-ERD response for sensorimotor test than for motor test (significant effect of SM/M in the R-ANOVA analysis (*p* < 0.001). **b** Beta-ERD was significantly increased preictally (*p* = 0.038)

Paired comparison (Table 2) revealed significantly increased beta-ERD responses in contralateral sensorimotor cortex (C3) in the preictal phase compared to the interictal phase for both the SM and M-tasks (C3 SM, *p* = 0.038; C3 M, *p* = 0.049; Fig. 3). However, R-ANOVA (Table 3) revealed only a trend towards a difference between the interictal and preictal phase (*p* = 0.089) presumably because the C4-response was similar in preictal and interictal phases (Table 2). Furthermore, paired Student’s t-tests for baseline (Table 4) revealed significantly increased beta power in contralateral sensorimotor cortex (C3) in the preictal phase compared to the interictal phase for both the SM and M-tasks (C3 SM, *p* = 0.007; C3 M, *p* = 0.048). We also discovered significantly increased baseline beta power (Table 4) in ipsilateral sensorimotor cortex (C4) in the ictal phase compared to the interictal phase for the SM test (C4 SM, *p* = 0.009).

**Table 4:** Baseline beta power for preictal-interictal, ictal-interictal and postictal-interictal

Interval mean	Interictal	Compared period	
C3 Sensorimotor			p
Preictal	1.27 (1.05–1.50)	1.34 (1.08–1.59)	**.007**
Ictal	1.23 (1.05–1.41)	1.24 (1.03–1.46)	.696
Postictal	1.23 (0.99–1.46)	1.21 (1.08–1.35)	.802
C3 Motor			
Preictal	1.28 (1.07–1.49)	1.33 (1.08–1.58)	**.048**
Ictal	1.25 (1.07–1.42)	1.25 (1.06–1.44)	.868
Postictal	1.26 (1.01–1.50)	1.25 (1.04–1.45)	.692
C4 Sensorimotor			
Preictal	1.31 (1.09–1.54)	1.34 (1.07–1.62)	.335
Ictal	1.23 (1.03–1.43)	1.28 (1.05–1.50)	**.009**
Postictal	1.26 (1.04–1.49)	1.25 (1.10–1.39)	.692
C4 Motor			
Preictal	1.32 (1.10–1.55)	1.34 (1.09–1.59)	.637
Ictal	1.27 (1.07–1.46)	1.28 (1.05–1.50)	.648
Postictal	1.29 (1.05–1.53)	1.28 (1.04–1.51)	.765

Beta power to the 0.25th power in the interval − 3 to −1 s before movement onset (baseline). Tabulated as mean ± SD. Post-hoc paired Student’s t-tests are included. EEG from central electrodes C3 (contralateral, left) and C4 (ipsilateral, right) for the sensorimotor test and motor test

### Interictal analyses between migraine patients and controls

Contralateral beta-ERD was more evident for the SM-task than for the M-task, both in migraine (SM test ratio 0.79) and controls (SM test ratio 0.82; Table 5). This was also shown by a significant effect of SM/M in the R-ANOVA analysis (*p* < 0.001; Table 6 and Fig. 3). R-ANOVA confirmed no overall difference in beta-ERD between migraine patients and controls (Table 6). For both groups we observed a generally stronger beta-ERD at the contralateral side (C3) revealed by a significant effect of side (Table 6). We found no significant differences in baseline beta power between interictal migraineurs and controls.

**Table 5:** Beta-ERD mean of response/baseline ratios in interictal migraine subjects and controls

Beta-ERD response	Mean ratio (± SD retransformed)				
	Migraine	Control	t	df	p
C3 SM	0.79 (0.54–1.16)	0.82 (0.51–1.32)	.30	57.4	.76
C3 M	0.99 (0.65–1.51)	0.88 (0.61–1.28)	−1.12	61.8	.27
C4 SM	0.77 (0.53–1.13)	0.87 (0.58–1.31)	1.21	60.6	.23
C4 M	1.05 (0.63–1.74)	1.07 (0.70–1.64)	.14	61.3	.89

Beta-ERD response is the ratio between mean power in the interval from 1 to 3 s after movement onset and mean power in the interval − 3 to −1 s before movement onset (baseline). Ratios were LN-transformed before statistical analysis and retransformed to mean ratios and mean ± SD for tabulation. Two-sample Student’s t-tests (equal variance not assumed) are included. EEG from central electrodes C3 (contralateral, left) and C4 (ipsilateral, right). *SM* = sensorimotor test, *M* = motor test

**Table 6:** Repeated measures ANOVA beta-ERD analysis of interictal migraine patients compared to controls

	Beta-ERD response	
Within subjects effect	F (1, 61)	p
Side	7.24	**.009**
SM/M	14.65	**<.001**
Side × SM/M	10.27	**.002**
Side × Group	3.94	**.052**
SM/M × Group	1.36	.25
Side × SM/M × Group	.40	.53
Between subjects effect	F (1, 61)	p
Group	.027	.87

Beta-ERD response is the ratio between mean power in the interval from 1 to 3 s after movement onset and mean power in the interval − 3 to − 1 s before movement onset (baseline). Ratios were LN-transformed before statistical analysis. Within subject factors used were side (C3 vs C4 electrode) and SM/M (sensorimotor vs motor task). The between subjects factor is Group (interictal migraine vs controls)

### Relationship between beta-ERD and clinical variables

In order to limit the number of estimations, correlation coefficients were only calculated from contralateral beta-ERD values in the interictal and preictal phases. No significant correlations were found for these variables. A trend was seen towards a positive correlation between preictal contralateral (C3) beta-ERD in the M-task and headache intensity (rho = 0.41, *p* = 0.095), and towards a negative correlation between interictal contralateral (C3) beta-ERD in the SM-task and headache history (rho = − 0.32, *p* = 0.076).

## Discussion

The main result in this study was increased beta-ERD in the preictal phase compared to the interictal phase over contralateral sensorimotor cortex. This finding suggests that cortical processing of sensorimotor input changes during 36 h before the headache attack in migraine. Based on current understanding of the beta-ERD property during hand movement [27, 28, 30], the findings suggest increased sensorimotor cortex excitability in the preictal phase compared to the interictal phase. Another important finding was the larger preictal baseline beta activity in migraine, possibly representing lower sensorimotor cortex pre-activation in the preictal phase. We did not find this central sensory fluctuation during the ictal phase, but the study was not powered to perform direct comparisons between the preictal and the ictal phase. Moreover, we did not discover any significant differences in baseline beta power or beta-ERD between migraineurs in the interictal phase and control subjects.

We observed generally larger strength of ERD during the sensorimotor test than during the pure motor test. The sensorimotor process measured as beta-ERD is averaged over time and consists of several sub-processes from different neuronal networks including motor intention, motor planning, command generation and sensory feedback. To execute movement over time it is necessary to maintain motor processing of proprioceptive sensory feedback. Studies on beta-ERD have shown the motor command-generating process to have little effect on the ERD response. Instead, ERD seems to reflect variation in proprioceptive sensation [30]. This theory might explain why the ERD response was larger for the sensorimotor task compared to the motor task. Accordingly, the observed cyclic fluctuation of beta-ERD in migraineurs may represent a relative change in central sensory processing.

The present results can be largely explained as an increased contralateral sensorimotor cortical response to movement in the preictal phase compared to the interictal phase. Increased ERD response may reflect increased cortical excitability. However, increased baseline beta power suggests increased preictal intracortical inhibition and/or less preictal thalamocortical activation prior to stimuli. The findings therefore indicate that cortical excitation during the task is increased relative to an altered baseline. We interpret this as reduced sensorimotor cortical pre-activation in the preictal phase.

Reduced pre-activation was originally proposed to reflect reduced serotenoergic “state setting” in order to explain intensity-dependence of auditory evoked potentials [39] and a possible VEP-habituation deficit interictally in migraine [40]. However, neither hyperexcitability, nor the preferred concept of “hyperresponsitivity” [41], have been confirmed in later blinded studies of interictal VEP [42, 43].

Cosentino et al. [23] found normal preictal potentiation of motor evoked potential trains (5 Hz MEP) in migraine while flat or inhibitory patterns were observed in interictal, ictal and postictal phases. This MEP-potentiation is thought to be mediated by cortical short-term synaptic enhancement mainly due to calcium-dependent regulation of glutamate release. Their results were interpreted as high (i.e. normal) threshold for inhibitory homeostatic responses in the preictal phase, due to low motor cortex activity. [23]. The same group suggested hyperresponsivity of excitatory intracortical circuits as the pathogenesis of migraine [44]. Compensatory effects such as lower thresholds for inhibitory regulation and increased “top-down” inhibitory control could explain normal interictal cortical activation despite this underlying cortical hyperresponsivity [44]. Destabilization of this excitatory/inhibitory balance could be the precipitating factor triggering the migraine attack. Decrease in cortical activation observed as increased beta activity in this study may cause higher thresholds for inhibitory homeostatic plasticity in the preictal phase as seen in the mentioned study of MEP-potentiation [23, 44]. Consequently, reduced inhibitory control of the suggested primitive hyperresponsivity might be represented as increased preictal beta-ERD as seen in this study.

In our previous article we found increased preictal PMBS in the ipsilateral sensorimotor cortex [14], suggesting increased inhibition 1–3 s after the termination of movement. Interneurons receiving facilitatory stimuli from interhemispheric projections [14, 45–47] may also be involved because PMBS-changes was mostly ipsilateral [13]. Hence, the combined results of increased preictal baseline-beta, beta-ERD and PMBS suggest that the sensorimotor cortex may undergo an increased deactivation-activation-deactivation cycle within the few seconds before, during and after the sensorimotor movement task. This may reflect an instability of thalamocortical, interhemispheric and intracortical regulatory mechanisms in the preictal phase.

Preictal cortical deactivation is in concert with recent findings of reduced baseline pain scores that suggested enhanced preictal endogenous analgesia [1]. Reduced habituation to electrically induced pain [48] and tonic heat pain [1] has been shown in the preictal phase of migraine, suggesting hyperresponsivity to persistent painful stimulation. Also, Marciszewski et al. found decreased pain sensitivity combined with greater activation of spinal trigeminal nucleus during noxious orofacial stimulation in the period immediately before a migraine attack [49].

Thalamus is probably also important in mediation of dysfunctional pain modulation in migraine, at least between attacks [50–52]. Our results show that there also is increased responsivity during non-noxious sensory stimuli at a thalamocortical level in the preictal phase. Meylakh et al. [53] recently reported increased power of infra-slow fMRI-oscillations in thalamus, PAG and hypothalamus immediately prior to the migraine attack. These findings hypothesized to reflect increased amplitude and synchrony of astrocyte calcium waves are difficult to compare with results based on electrophysiological recordings. However, recent longitudinal fMRI studies also suggest that a hypothalamic-brainstem network dysfunction may be relevant during the preictal phase of migraine [11].

We did not detect significant differences in baseline beta power or beta-ERD between migraineurs in the interictal phase and control subjects. Reduced interictal cortical pre-activation has been proposed previously, mainly based on a non-significant trend towards lower first-block visual evoked potential (VEP) amplitude in migraineurs [40, 54, 55]. However, first-block VEP amplitude is normal in most studies [42, 43]. Hence, neither previous VEP nor present beta-ERD results support this theory for the interictal phase in migraine. Furthermore, the theory of cortical hyperresponsivity compensated interictally by inhibitory control in migraine could be an explanation of non-significant interictal analyses. However, it is also possible that increased pre-activation variability is present interictally, explaining why results differ between studies.

### Strengths and limitations of the study

It is possible that many of the neurophysiological changes in the migraine brain are subtle, needing higher-powered studies to be consistently observed. Furthermore, a great challenge in evaluating the migraine cycle is to conduct longitudinal studies with enough subjects for paired recordings, and a greater number of subjects with more test repetitions can detect smaller changes. Small groups for paired analyses may have contributed to the possibility of type II errors in this study. However, a blinded, longitudinal study design such as in the present study is important to investigate the very phase specific alterations of migraine pathophysiology [56].

Furthermore, healthy subjects were not screened for relatives with migraine, and a few controls might in theory be susceptible to migraine and to VEP-potentiation [57]. Moreover, in a previous sensitivity analysis the removal of controls with migraine relatives did not affect the VEP habituation results [43]. This potential confounder may possibly contribute to the risk of type II errors, but it should not affect present positive beta-ERD findings.

Scalp electrical recordings is known to be contaminated by contributions of electromyography, possibly from activation of scalp and neck muscles [58]. The present study did not utilise specific methodology for reducing such artefacts. However, EMG contamination is mostly shown for frequencies above 20 Hz which is higher than the frequency band chosen for our beta power analyses [58].

In studies of event related dynamics, it is important to differentiate between self-paced and triggered movement as neurophysiological changes may be detected before the movement itself when there is a planning phase [29]. All movements in this study was triggered to limit the uncertainty in determining start of relevant cortical activity during a planning phase.

As in our previous study [14], an a priori methodological selection of the interval 12–19 Hz as beta band [35, 36] was conducted to avoid type I errors. However, other studies on beta event related dynamics have yielded results for different cut-offs for beta band frequencies [59–62]. It is possible that identical oscillatory processes are reflected by slightly different beta band frequencies between subjects. Such interpersonal differences may affect results from two-sample statistics comparing groups, however the significant findings in this study was found for longitudinal analyses of the same subjects. Furthermore, more recent findings indicate that movement-related beta oscillations show high intraindividual reliability and are well suited to detect individual differences in a longitudinal study design [63].

The present ERD-calculation method does not differentiate between phase-locked and non-phase-locked activity [27], but this is seemingly not necessary for movement related beta-alterations [64]. For future studies it would be of interest also to try more advanced time-frequency domain methods like event related spectral perturbation (ERSP) also for movement related ERD-paradigms [65].

## Conclusion

We were able to show cyclic fluctuation of cortical sensory processing in migraine patients through alterations in beta-ERD and baseline beta power. In the 36-h period before headache, there seems to be a decrease in cortical pre-activation and an increase in the relative stimulus-induced response. Altered pre-activation may lead to changes in thresholds for inhibitory responses and consequently increased beta-ERD response, possibly reflecting a generally increased cortical responsivity in migraine. We did not find this central sensory fluctuation during the ictal phase, but the study was not powered to perform direct comparisons between the preictal and the ictal phase. Cyclic alterations in the activity of second- and third-order afferent somatosensory neurons, and their associated cortical and/or thalamic interneurons, may accordingly be a central part of the migraine pathophysiology. Combined investigations of cyclic fluctuations in event related responses (ERD/ERS) and targeted transcranial magnetic stimulation (TMS) paradigms should be conducted to understand the involvement of different excitatory and inhibitory pathways in migraine pathophysiology.

## Data Availability

The datasets used and analysed during the current study are available from the corresponding author on reasonable request.

## Abbreviations

CNS: Central Nervous System
ECG: Electrocardiography
EEG: Electroencephalography
EMG: Electromyography
ERD: Event-related desynchronization
ERS: Event-related synchronization
GABA: Gamma-Aminobutyric acid
M: Motor test
MEP: Motor evoked potentials
NMDA: N-Methyl-D-aspartate
PAG: Periaqueductal gray
PMBS: Post-movement beta synchronization
R-ANOVA: Repeated measures ANOVA
SM: Sensorimotor test
TMS: Transcranial Magnetic Stimulation
VEP: Visual evoked potentials
